# Individual Watershed Areas in Sickle Cell Anemia: An Arterial Spin Labeling Study

**DOI:** 10.3389/fphys.2022.865391

**Published:** 2022-05-03

**Authors:** Hanne Stotesbury, Patrick W. Hales, Anna M. Hood, Melanie Koelbel, Jamie M. Kawadler, Dawn E. Saunders, Sati Sahota, David C. Rees, Olu Wilkey, Mark Layton, Maria Pelidis, Baba P. D. Inusa, Jo Howard, Subarna Chakravorty, Chris A. Clark, Fenella J. Kirkham

**Affiliations:** ^1^ Imaging and Biophysics Section, Developmental Neurosciences, UCL Great Ormond St. Institute of Child Health, London, United Kingdom; ^2^ Division of Psychology and Mental Health, Manchester Centre for Health Psychology, University of Manchester, Manchester, United Kingdom; ^3^ Radiology, Great Ormond Hospital for Children NHS Foundation Trust, London, United Kingdom; ^4^ Paediatric Haematology, King’s College Hospital NHS Foundation Trust, London, United Kingdom; ^5^ Paediatric Haematology and Oncology, North Middlesex University Hospital NHS Foundation Trust, London, United Kingdom; ^6^ Haematology, Imperial College Healthcare NHS Foundation Trust, London, United Kingdom; ^7^ Department of Haematology and Evelina Children’s Hospital, Guy’s and St Thomas’ NHS Foundation Trust, London, United Kingdom; ^8^ Clinical Neurosciences Section, Developmental Neurosciences, UCL Great Ormond St. Institute of Child Health, London, United Kingdom

**Keywords:** MRI, arterial spin labeling, silent cerebral infarction, cerebral hemodynamics, hemoglobinopathies, cognition, intelligence quotient (IQ), processing speed index

## Abstract

Previous studies have pointed to a role for regional cerebral hemodynamic stress in neurological complications in patients with sickle cell anemia (SCA), with watershed regions identified as particularly at risk of ischemic tissue injury. Using single- and multi-inflow time (TI) arterial spin labeling sequences (ASL) in 94 patients with SCA and 42 controls, the present study sought to investigate cerebral blood flow (CBF) and bolus arrival times (BAT) across gray matter, white matter with early arrival times, and in individual watershed areas (iWSAs). In iWSAs, associations between hemodynamic parameters, lesion burden, white matter integrity, and general cognitive performance were also explored. In patients, increases in CBF and reductions in BAT were observed in association with reduced arterial oxygen content across gray matter and white matter with early arrival times using both sequences (all *p* < 0.001, d = −1.55–−2.21). Across iWSAs, there was a discrepancy between sequences, with estimates based on the single-TI sequence indicating higher CBF in association with reduced arterial oxygen content in SCA patients, and estimates based on the multi-TI sequence indicating no significant between-group differences or associations with arterial oxygen content. Lesion burden was similar between white matter with early arrival times and iWSAs in both patients and controls, and using both sequences, only trend-level associations between iWSA CBF and iWSA lesion burden were observed in patients. Further, using the multi-TI sequence in patients, increased iWSA CBF was associated with reduced iWSA microstructural tissue integrity and slower processing speed. Taken together, the results highlight the need for researchers to consider BAT when estimating CBF using single-TI sequences. Moreover, the findings demonstrate the feasibility of multi-TI ASL for objective delineation of iWSAs and for detection of regional hemodynamic stress that is associated with reduced microstructural tissue integrity and slower processing speed. This technique may hold promise for future studies and treatment trials.

## Introduction

Sickle cell anemia (SCA) is one of the most prevalent and severe monogenic disorders, with an estimated 20–25 million people affected worldwide (Aygun and Odame, 2012). Historically, SCA was largely characterised by episodes of acute illness, with pain crises often referred to as the “hallmark” complication (Ellison and Shaw, 2007), and interim periods of relative health as “steady-state” periods. However, with increasing life-expectancy in high-income countries (Gardner et al., 2016), chronic complications resulting from progressive end-organ disease are increasingly described (Chaturvedi et al., 2018).

Among the most common complications are those affecting the brain, including overt stroke, silent cerebral infarction (SCI), reduction in microstructural white matter integrity, and cognitive impairment (Prussien et al., 2019; Stotesbury et al., 2021). In recent years, evidence that cerebral hemodynamic stress may play a role in brain complications has been accumulating (Jordan and DeBaun, 2018; Stotesbury et al., 2019; Wang et al., 2021). However, several unique and interdependent features of SCA pathology pose considerable challenges to understanding the nature of this role, including a right-shifted oxygen dissociation curve (Milner, 1974; Needleman et al., 1999), altered red cell rheology (Connes et al., 2016; Brousse et al., 2021), reduced oxygen-carrying capacity, and abnormal cerebral blood velocity and flow (CBF) (Behpour et al., 2013; Jordan and DeBaun, 2018).

It is relatively well established that global gray matter CBF is on average elevated in “steady-state” patients with SCA. Elevated CBF occurs in the context of reduced arterial oxygen content (Bush et al., 2016; Stotesbury et al., 2022), indicative of a compensatory mechanism, which appears to maintain oxygen delivery when averaged globally (Chai et al., 2019), but is associated with reduced cerebrovascular reserve (Nur et al., 2009; Prohovnik et al., 2009; Kim et al., 2016; Kosinski et al., 2017; Václavů et al., 2019) and abnormal oxygen extraction fraction (OEF) (Jordan et al., 2016; Bush A. M. et al., 2018; Fields et al., 2018; Vaclavu et al., 2018; Watchmaker et al., 2018). Although several studies also indicate that global white matter CBF is on average elevated (Helton et al., 2009; Fields et al., 2015, 2018; Stotesbury et al., 2022), the elevation appears to be lower than that observed for gray matter, and may therefore be insufficient to maintain oxygen delivery during acute illness (e.g., acute anemic events, acute chest syndrome), particularly in the deep watershed white matter, where vascular supply and CBF are already inherently low (Stotesbury et al., 2018a, 2019).

Results from one relatively recent study in adolescents and young adults are consistent with this notion, and suggest that global white matter oxygen delivery is significantly reduced even in “steady-state” patients compared to controls (Chai et al., 2019). Using arterial spin labeling (ASL), the authors found significantly elevated global gray matter CBF in patients, but no differences in global white matter CBF, potentially indicating inadequate compensatory vasodilation in white matter. Importantly, through t-score maps, the authors showed the reduction in white matter oxygen delivery to be disproportionate in regions corresponding visually with the deep watershed regions that appear to be particularly vulnerable to SCI and microstructural injury (Chai et al., 2019). Taken together, these findings are consistent with the “perfusion paradox” that has been described in other vascular beds, with hyper-perfusion in the macro-circulation, and relative hypo-perfusion in the microcirculation (Nath et al., 2004).

Further indicative of regional vulnerability to hemodynamic stress, two recent studies showed considerable overlap between the areas of highest SCI density and lowest CBF (Ford et al., 2018) and highest OEF (Fields et al., 2018) in independent SCA cohorts. The “at-risk” regions identified in both studies again corresponded visually with the deep watershed regions. Both studies used the traditional pediatric Silent Infarct Transfusion Trial definition of SCI, requiring an area of abnormally high signal intensity on T2-weighted and/or fluid-attenuated inversion recovery (FLAIR) magnetic resonance imaging (MRI), measuring at least 3 mm in greatest dimension, visible on two planes, and with no corresponding focal neurological deficit. The studies also respectively based their SCI density map on low-resolution images from the Silent Infarct Transfusion Trial (Ford et al., 2018) or a combination of low- and high-resolution images performed for clinical care over a 10-year period (Fields et al., 2018). Studies relying on lower field strength magnets (i.e. 1.5T) and lower-resolution sequences (i.e. 3-5 mm slice thicknesses) are by definition likely to miss some lesions meeting traditional SCI criteria, and there is evidence that lesion detectability increases with increasing magnetic field strength (van der Land et al., 2015b). Moreover, both studies used manual delineation of lesions and both thresholded their density maps, either to include SCIs from 90% of the cohort, or to include only areas of SCI occurring in two or more participants. It is therefore likely that smaller white matter hyperintensities, and/or hyperintensities occurring in less commonly affected regions, were excluded and/or missed.

Further, prior hemodynamic MRI studies in patients with SCA have primarily used phase-contrast imaging (Vu et al., 2021), dynamic susceptibility contrast imaging (Kirkham et al., 2001) or ASL data collected at a single inflow time (single-TI, Bush A. et al., 2018; Chai et al., 2019; van den Tweel et al., 2009). Whilst dynamic susceptibility contrast imaging requires injection of a gadolinium-based contrast agent, and phase-contrast imaging only allows for whole-brain estimation of CBF in major arteries, single-TI ASL techniques allow for non-invasive estimation of CBF on a voxel-wise basis but rely on a number of assumptions. In single-TI ASL, radiofrequency pulses are applied to magnetically label blood water in the cervical arteries. After a few seconds (the inflow time, TI) the magnetically labeled blood water flows into and exchanges with brain tissue water. An image is then acquired and subtracted from a control image with no labeled blood water. The signal difference is used to provide an estimate of the amount of blood that has been delivered at that particular inflow time.

Although there have been relatively few studies in clinical populations, multi-TI ASL techniques eliminate the need for assumptions about arterial transit times by acquiring images at multiple TIs, allowing the passage of labeled blood water to be fully quantified on a voxel-wise basis (Buxton et al., 1998). In the multi-TI studies that have been conducted to date, patients with SCA have been shown to exhibit increased CBF and reduced bolus (i.e. blood) arrival times (BAT) across gray (Juttukonda et al., 2017; Kawadler et al., 2018; Stotesbury et al., 2022) and white matter (Stotesbury et al., 2022). Interestingly, across high-signal regions of the sagittal sinus, both CBF and BAT have been found to be increased (Stotesbury et al., 2022), potentially reflecting increased resistance in the microcirculation secondary to abnormal erythrocyte rheology (Stotesbury et al., 2022) and functional shunting (Østergaard et al., 2016; Juttukonda et al., 2019). Using multi-TI ASL, increased CBF has also been associated with worse cognitive performance across white matter and high-signal sagittal sinus regions, but not across gray matter (Stotesbury et al., 2022).

Theoretically, BAT maps can be used to extract, physiologically rather than visually, individual watershed white matter regions (iWSAs) with late inflow times. Despite accumulating evidence indicating that these regions may be particularly vulnerable to hemodynamic stress, SCI, and microstructural injury in several populations with cerebrovascular disease, including SCA, iWSAs have so far primarily been explored using dynamic susceptibility contrast imaging techniques in elderly populations with carotid artery stenosis (Kaczmarz et al., 2021). This is in part because ASL still suffers from relatively low signal to noise ratio, and the signal in deep white matter may be unreliable. Low signal to noise ratio is however likely to be less of an issue in populations with higher CBF, such as healthy children and young adults, as well as patients with SCA of all ages. Moreover, it is possible to identify and remove voxels with unreliable signal prior to analysis (Wu et al., 2013).

Building on this work, the purpose of this study was therefore to use both single-TI and multi-TI ASL to compare CBF and BAT in relation to arterial oxygen content in three regions of interest (ROIs) in patients with SCA and controls; gray matter, white matter with early arrival times, and for the first time using ASL, in iWSAs. Based on the aforementioned studies, we hypothesised that while CBF would be increased and BAT reduced in grey matter and white matter with early arrival times in patients with SCA, there would be no between group differences in iWSAs. Lesions burden in iWSAs was also compared between patients and controls, and associations between hemodynamic parameters, lesion burden, white matter integrity and cognitive outcome were explored in this region.

## Methods

### Participants

Patients were recruited to two concurrent studies with overlapping MRI and cognitive assessment protocols between 2015 and 2019: the Sleep Asthma Cohort follow-up (SAC) (Rosen et al., 2014) and the Prevention of Morbidity in Sickle Cell Anemia baseline investigation (POMS) (Howard et al., 2018). Controls were siblings and race-matched peers (i.e. Black British) of patients recruited to SAC. Patients were ineligible for POMS study participation if they were receiving nocturnal respiratory support at the time of enrollment, participating in a clinical trial evaluating blood transfusion or oxygen therapy, or had chronic lung disease (other than asthma) or existing respiratory failure. Additional exclusion criteria for the POMS study were hospital admissions for acute sickle complications within 1 month of enrollment, more than six hospital admissions for acute sickle complications within 12 months of enrollment, overnight oximetry showing mean overnight saturation of less than 90% for more than 30% of total sleep time, severe sleep apnea defined by 4% oxygen desaturation index >15/h, and chronic blood transfusion or transfusion within 3 months of enrollment. For the SAC study, patients were enrolled without regard to past sickle- or sleep-related morbidity or transfusion status. We have previously described average ASL parameters across grey matter, white matter, and high signal regions of the sagittal sinus in this sample of participants (Stotesbury et al., 2022).

### Cognitive and Socio-Economic Measures

Intelligence quotient (IQ) was estimated using the two-subtest Wechsler Abbreviated Scale of Intelligence (WASI; POMS participants, Wechsler, 2013), the Wechsler Intelligence Scale for Children (WISC-IV; SAC participants <16 years, Wechsler, 2008), or the Wechsler Adult Intelligence Scale (WAIS-IV; SAC participants >16 years, Wechsler, 2008). Subtests from the WISC-IV or WAIS-IV measuring working memory and processing speed were used to calculate composite indices (Working Memory Index and Processing Speed Index, respectively). Participants were assessed as close to the date of MRI as possible, with 76% undergoing both on the same day, and all undergoing both within 4.5 months.

### Hematological Measures and Treatment

In patients, hydroxycarbamide/hydroxyurea use, chronic transfusion regimens, and the closest routine full blood count to date of MRI were collected from relevant medical records. In controls, hematocrit and hemoglobin were estimated based on age and sex using appropriate literature values (Castro et al., 1987). Peripheral oxygen saturation was recorded using a pulse oximeter. For four patients with missing hemoglobin, and seven controls with missing oxygen saturation, group means were substituted. Assuming pO2, the partial pressure of oxygen, is 100 Torr in alveoli and arterial blood in subjects breathing room air, arterial oxygen content was calculated as:

(1.34 × Hemoglobin (g/dl)×%Oxygen Saturation)+(0.003 × pO_2_)

### MRI Acquisition

MRI was performed on a 3T Siemens Prisma (Erlangen, Germany) MRI scanner with 80mT/m gradients and a 64-channel receive head coil at Great Ormond Street Children’s Hospital. The protocol included:

• a prototype single-TI pseudo-continuous ASL (pCASL) acquisition, with background suppression, and a 3D gradient-and-spin-echo readout (repetition time (TR) = 4,620 ms, echo time (TE) = 21.8 ms, labeling duration = 1800 ms, post-labeling delay = 1500 ms, repetitions = 10, field of view = 220 mm, matrix size = 64 × 62, in-plane resolution = 1.7 × 1.7 mm (after x2 zero-filling), number of partitions = 24, slice thickness = 4.0 mm, turbo factor = 12, echo-planar imaging (EPI) factor = 31, segments = 2, with parallel imaging, generalized autocalibrating partial parallel acquisition (GRAPPA) acceleration factor = 2). A proton-density weighted (M0) image was also acquired (TR = 4000 ms), with specifications identical to the pCASL acquisition but with the labeling pulses removed. Total acquisition time was 3 min 19 s.
• a prototype multi-TI pulsed ASL (PASL) acquisition, with background suppression, and a 3D gradient-and-spin-echo readout (10 inflow times with one acquisition per TI, ranging 350–2600 ms in 250 ms intervals, and TR of 3,300 ms; all other readout parameters were identical to the pCASL acquisition. Q2TIPS (Luh et al., 1999) RF pulses were applied 700 ms after the labeling pulse to define the temporal width of the bolus. As for the single-TI acquisition, a proton-density weighted map was acquired (TR = 4000 ms) with labeling removed. Total acquisition time was 2 min 25 s
• an axial diffusion-weighted 2D spin-echo multi-shot acquisition with echoplanar imaging readout (TR = 3,050 ms, TE = 60 ms, 2 shells at b = 1,000 s/mm2 and b = 2,200 s/mm2 with 60 non-collinear diffusion directions per shell and 13 interleaved b = 0 images, voxel size = 2 × 2 × 2 mm)
• a coronal high-resolution 3D FLAIR sequence (TR = 5000 ms, TE = 395 ms, voxel size = 0.65 × 0.65 × 1.0 mm, scan time = 6 min 22 s),
• an axial 2D T2-w turbo spin echo sequence (TR = 8420 ms, TE = 68 ms, voxel size = 0.50 × 0.50 × 4.0 mm, scan time = 2 min, 50 s)
• a T1-w magnetization-prepared rapid acquisition gradient echo sequence (TR = 2,300 ms, TE = 2.74 ms, TI = 909 ms, flip angle = 8°, voxel size = 1 × 1 × 1 mm, scan time = 5 min, 21 s)
• 3D time-of-flight magnetic resonance angiography (TR = 21.0 ms, TE = 3.4 ms, scan time = 5 min, 33 s).

#### Radiological Measures

A neuroradiologist (D.S.) diagnosed lesions as regions of abnormally high signal intensity, consistent with ischemia, visible in two planes on FLAIR and T2-weighted MRI, in the absence of overt focal neurological symptoms. Clearly distinguishable lesion mimics (e.g. perivascular spaces) were excluded. Generous masks were manually drawn around the identified lesions on native FLAIR images, before FLAIR and T1-weighted images were bias-corrected using Statistical Parametric Mapping (Wellcome Trust Centre for Neuroimaging, London, UK; http://www.fil.ion.ucl.ac.uk/spm), and linearly affine-aligned with nearest neighbour interpolation using the FMRIB Software Library (FSL, Oxford, UK; https://fsl.fmrib.ox.ac.uk/fsl/fslwiki/FSL). As in prior studies (van der Land et al., 2015a), for each participant the mean FLAIR intensity across the cortex was calculated, and a lower threshold was used to determine which voxels within lesion ROIs should be included in a lesion burden mask:

(1) LowerThresholdflair = 1.02 x meanFLAIRcortex

#### Hemodynamic Parameters

As described in prior ASL studies in this cohort (Stotesbury et al., 2022), raw single-TI and multi-TI ASL images were inspected, and excluded if there were major motion-related artifacts. As ASL model fitting requires a value representing the T1 relaxation rate of blood (T1_bl_), and T1_bl_ is dependent on hematocrit and oxygen saturation, estimated values were calculated based on measured (patients) or estimated (controls) hematocrit and measured oxygen saturation (Hales et al., 2016).

In-house MATLAB scripts were used to pre-process the raw ASL images. Single-TI images were co-registered using the FSL *flirt* tool (version 6.0; FMRIB, Oxford, UK; https://fsl.fmrib.ox.ac.uk/), with affine registrations (12 degrees of freedom) derived from a correlation ratio cost function. The mean single-TI pCASL difference signal was calculated over the 10 co-registered repetitions, and used to estimate CBF following the method described in Alsop et al. (2015), with *λ* = 0.9, α = 0.85, τ = 1.8, and patient-specific estimates of T1_bl_ (mean SCA = 1.99 s, standard deviation, sd SCA = 0.09 s, mean control = 1.77 s, sd control = 0.07 s).

For the multi-TI images, where motion occurred at later TIs (>1.35 s), images were co-registered using the FSL flirt tool, with affine registrations as described for the single-TI images. Where motion occurred at earlier inflow times with lower signal to noise ratio, datasets were excluded. To remove voxels with unreliable signal, four square-shaped ghost-free background ROIs with a width of 5 voxels were created and placed in each corner of the difference image timeseries. The mean and standard deviation of ghost-free background were computed. Reliable voxels were defined as those where the difference signal was at least greater than the mean + one standard deviation of ghost-free background on at least one image in the timeseries (Wu et al., 2013). The PASL difference signal was used to fit voxel-wise values of CBF and BAT using the general kinetic model described in Buxton et al. (1998), with *λ* = 0.9, α = 0.98, and τ = 0.7, and patient-specific estimates of T1_bl_ (values as for single-TI above).

### Microstructural Parameters

Diffusion weighted images were visually inspected, and excluded in the event of major motion-related artifacts. Diffusion data were then pre-processed using MRtrix3 (version 3.0; https://www.mrtrix.org) (Tournier et al., 2019). To denoise the data, the MRtrix3 *dwidenoise* tool was applied as the first step of pre-processing. Next, the MRtrix3 *mrdegibbs* tool was applied to remove Gibbs ringing artifacts (Kellner et al., 2016). Susceptibility-induced distortions and eddy current artifacts were then corrected using the MRtrix3 *dwipreproc* tool, which includes wrappers to FSL tools such as *topup* and *eddy* (Smith et al., 2004). Finally, a B1 field inhomogeneity correction was applied using the MRtrix3 *dwibiascorrect* tool (Kellner et al., 2016). To obtain fractional anisotropy parameter brain maps, a brain mask was obtained using the MRtrix3 *dwi2mask* tool, then voxel-wise diffusion tensor fitting was performed using the MRtrix3 *dwi2tensor* tool.

### Regions of Interest

Fractional anisotropy maps, the mean of the unlabeled single-TI images, and the multi-TI image with the longest inflow time (i.e. *TI =* 2600 ms) were aligned with T1w images using ANTs rigid and deformable transforms (i.e. antsRegistrationSyn; https://github.com/ANTsX/ANTs) with default settings.

**FIGURE 1:**
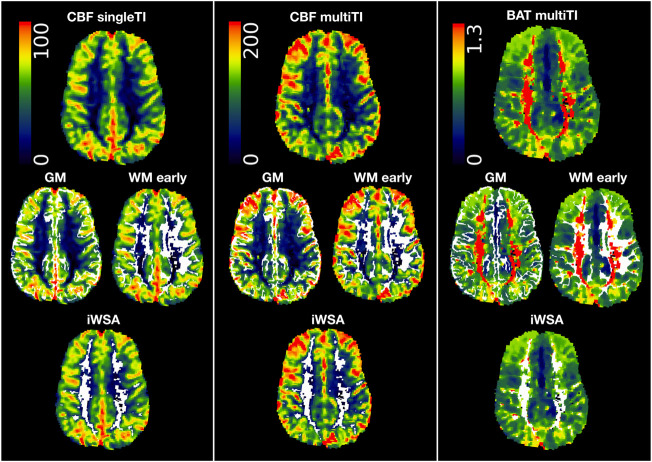
Regions of interest. Showing regions of interest (i.e., grey matter (GM), white matter with early arrival times (WM early), white matter with late arrival times/individual watershed areas (WM late, iWSA) overlaid in white on cerebral blood flow (CBF) and bolus arrival time (BAT) maps from the single inflow time (SingleTI) and multi inflow time (multiTI) sequences in a representative participant with sickle cell anemia (male, 12 years of age).

**FIGURE 2:**
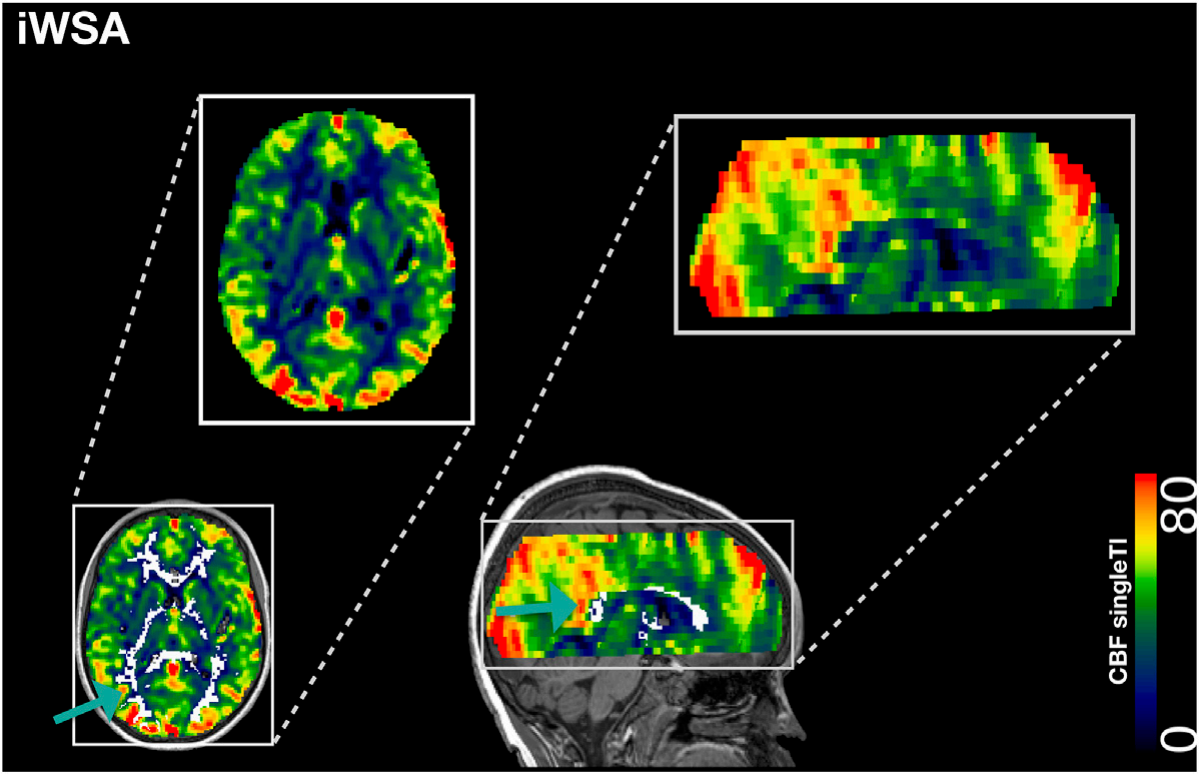
Individual Watershed Area. Showing the individual watershed area (iWSA) region of interest overlaid in white on a single inflow time cerebral blood blow (CBF) map from a representative participant with sickle cell anemia (male, 8 years of age).

To create binary masks for gray and white matter ROIs, cortical reconstruction and volumetric segmentation were performed on T1-w images using Freesurfer (Center for Biomedical Imaging, Massachusetts, USA; http://surfer.nmr.mgh.harvard.edu/). To correct for potential partial volume effects, gray and white matter masks were eroded using a kernel box with a width of 2 voxels in FSL. To extract iWSAs, a lower threshold corresponding to the 70th percentile of voxels within the eroded white matter masks was applied to each participant’s BAT map (Figure 1, Figure 2). Remaining voxels within the eroded white matter masks were then used to generate the early arrival time white matter ROI (Figure 1).

### Statistical Analysis

Mean CBF and BAT values were extracted across ROIs for both sequences and, after assessing normality (Shapiro-Wilk test), compared between patients with SCA and controls using Student’s t-tests for normally, and Mann-Whitney U tests for non-normally, distributed variables on a region-by-region basis. Multiple comparisons were corrected for using the false discovery rate procedure (Benjamini and Hochberg, 1995) within groups (i.e., patients, controls) and across parameter maps (i.e., single-TI CBF, multi-TI CBF, and multi-TI BAT).

Lesion load in iWSAs was compared between patients with SCA and controls using Mann-Whitney U tests. Relationships between age- and sex-adjusted CBF across iWSAs and lesion burden, age- and sex-adjusted iWSA FA, and cognitive performance were explored using Spearman’s Rho for non-normally, and Pearson’s correlations for normally, distributed variables. For any variables that were significantly associated with cognitive performance in patients or controls, further exploratory regression models were computed in the relevant group. Based on prior studies (Stotesbury et al., 2018b), pre-selected covariates comprising iWSA fractional anisotropy, lesion volume, arterial oxygen content, age, sex, total intracranial volume, and education deciles, were included in all models, and model assumptions and variance inflation factors were assessed.

For any hemodynamic parameter that was significantly associated with both microstructural integrity (i.e. fractional anisotropy) across iWSAs and cognitive outcome, exploratory causal mediation analysis was performed, with the relevant hemodynamic factor as the independent variable, and iWSA fractional anisotropy as the mediator. Age and sex were included as covariates. The significance of the indirect mediation effect was tested using bootstrapping procedures in the R package “mediate” (Tingley et al., 2014). Unstandardised indirect mediation effects were computed for each of 1,000 bootstrapped samples, and the 95% confidence interval was computed by determining the indirect effects at the 2.5th and 97.5th percentiles.

Across all analyses, a probability threshold of <0.05 was considered statistically significant. Effect sizes were estimated using Kendall’s W(w), Cramér’s-phi (φ), Cohens d (d), and correlation coefficients (r), and interpreted using Cohen’s conventions (small d = 0.2, w/φ/r = 0.1, medium d = 0.5,/w/φ/r/ = 0.3, large d = 0.8, w/φ/r = 0.5) (Cohen, 1988).

## Results

### Participants

**FIGURE 3:**
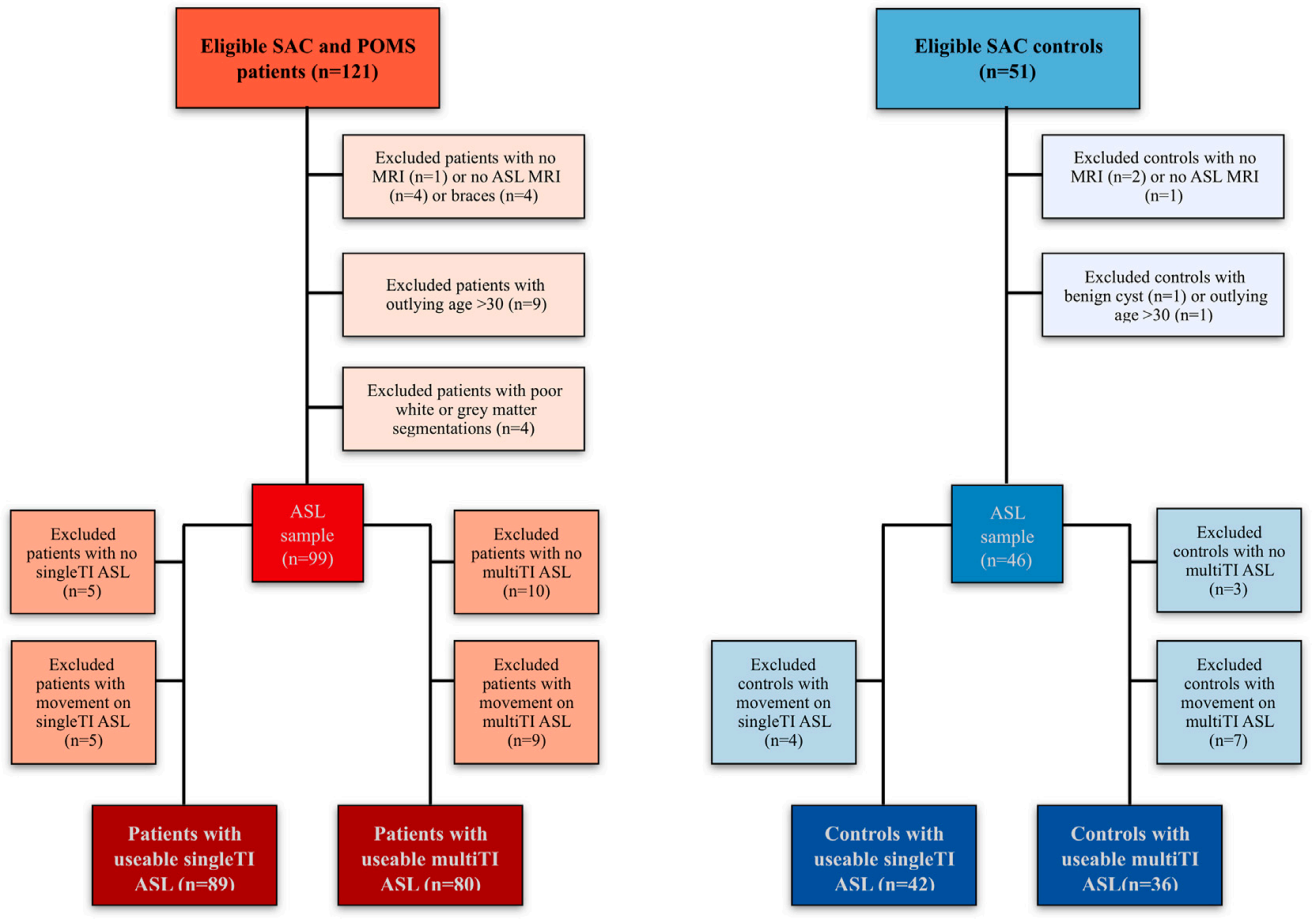
Participant flow-chart. Visualization of reasons for participant exclusion (Stotesbury et al., 2022). Note that while most participants had usable data for both sequences (N = 75 patients, *n* = 36 controls), others only had usable data for the single TI sequence (*n* = 14 patients, *n* = 6 controls), or the multiTI sequence (*n* = 5 patients, 0 controls). ASL Arterial Spin Labeling; MRI magnetic resonance imaging; POMS, Prevention of Morbidity in Sickle Cell Disease; SAC, Sleep Asthma Cohort; TI, inflow time.

As described in prior ASL studies in this cohort (Stotesbury et al., 2022), of 172 recruited participants, 94 patients (aged 8–27 years, 46 male, 93 homozygous for hemoglobin S (HbSS), 1 HbSb_0_-thalassemia) and 42 controls (age 8–30 years, 16 male, 26 siblings, 23 HbAA, 2 HbAC, 17 HbAS/sickle cell trait) had useable single-TI and/or multi-TI ASL data (Figure 3). Of the included participants, three patients had no cognitive data, seven patients received chronic blood transfusion, and 34 were prescribed hydroxycarbamide.

Lesions were detected in 45 patients and 10 controls, while 2 patients had large vessel vasculopathy (1 right internal carotid narrowing, 1 right middle cerebral artery stenosis). Arterial oxygen content was lower in patients with SCA compared to estimated values in controls, but there were no differences between patients and controls in age, sex, or education deciles (Table 1).

**TABLE 1:** Sample demographics. Values are summary and test statistics for all included participants with usable single and/or multi inflow time ASL data. Abbreviations: SCA, sickle cell anemia; sTI, single inflow time sequence; mTI, multi inflow time sequence; CBF, cerebral blood flow; BAT, bolus arrival time; iWSA, individual watershed areas; SD, standard deviation; IQR, interquartile range; p, probability values for between-group differences. Significant between-group differences are shown in bold.

	SCA (*n* = 94)		Control (*n* = 42)	between-group differences
Demographic Variables	Count (percentage)/Median (IQR)			
Sex	46 Male (48.94%)		16 Male (38.10%)	*p* = 0.32, *φ =* 0.08
Age (yr)	16.67 (13.32–19.89)		17.33 (14.57–20.48)	*p* = 0.65, r = 0.04
Education Decile	5 (4–7)		5 (4–6)	*p* = 0.72, r = 0.03
Hematological Variables	11.79 (10.39–12.92)	17.69 (17.42–18.22)		***p* < 0.0001, r = 0.79**
Arterial oxygen content (CaO_2,_ mL/d**)**				
Radiological Variables				
Lesions	45	10		***p* = 0.0005, *φ =* 0.21**
Lesion burden (1 mm^3^ voxels)	75 (26–188.0)	15 (4–34.5)		***p* = 0.006, 0.24**
Cognitive Variables	Mean (SD)/Median (IQR)			
Intelligence quotient (IQ)	92.63 (13.44)		98.10 (12.00)	***p* = 0.02, d = 0.43**
Working memory index (WMI)	91.73 (13.69)		99.24 (13.48)	***p* = 0.004, d = 0,55**
Processing speed index (PSI)	89.49 (12.96)		97.55 (13.17)	***p* = 0.001, d = 0.62**
Hemodynamic Variables	Mean (SD)/Median (IQR)			
*sTI ASL*	*n = 89*		*n = 42*	
Grey Matter CBF (ml/100 g/min)	54.06 (7.78)		42.52 (7.13)	***p* < 0.0001, d = -1.55**
White Matter—earlier arrival CBF (ml/100 g/min)	34.12 (4.82)		26.08 (4.85)	***p* < 0.0001, d = -1.66**
White Matter—iWSA CBF (ml/100 g/min)	24.11 (4.41)		16.85 (3.95)	***p* < 0.0001, d = -1.73**
*mTI ASL*	*n = 80*		*n = 36*	
Grey Matter CBF (ml/100 g/min)	129.02 (22.26)		89.62 (11.83)	***p* < 0.0001, d = -2.21**
White Matter—earlier arrival CBF (ml/100 g/min)	73.80 (14.55)		49.52 (8.39)	***p* < 0.0001, d = -2.04**
White Matter—iWSA CBF (ml/100 g/min)^a^	59.54 (14.61)		55.13 (13.14)	*p* = 0.11, d = -0.32
			
Grey Matter BAT (s)	0.72 (0.66–0.75)		0.82 (0.77–0.89)	***p* < 0.0001, r = 0.53**
White Matter—earlier arrival BAT (s)	0.74 (0.70–0.80)		0.85 (0.82–0.92)	***p* < 0.0001, r = 0.50**
White Matter—iWSA BAT (s)	1.51 (1.36–1.69)		1.86 (1.71–2.05)	***p* < 0.0001, r = 0.53**

a descriptive statistics and between-group differences in iWSA CBF reported with outlying control (Figure 5) excluded. Note that although including the outlier skews the mean in controls (m = 57.24, sd = 18.14), between-group differences remain non-significant (*p* = 0.51).

Cognitive performance was reduced in patients compared to controls, with average Wechsler scores 5 IQ points, 7 Working Memory Index points and 8 Processing Speed Index points lower than controls (all *p* < 0.05; Table 1). Cognitive performance in controls did not significantly differ as a function of sickle cell trait (all *p* > 0.05).

### Hemodynamic Parameters

**FIGURE 4:**
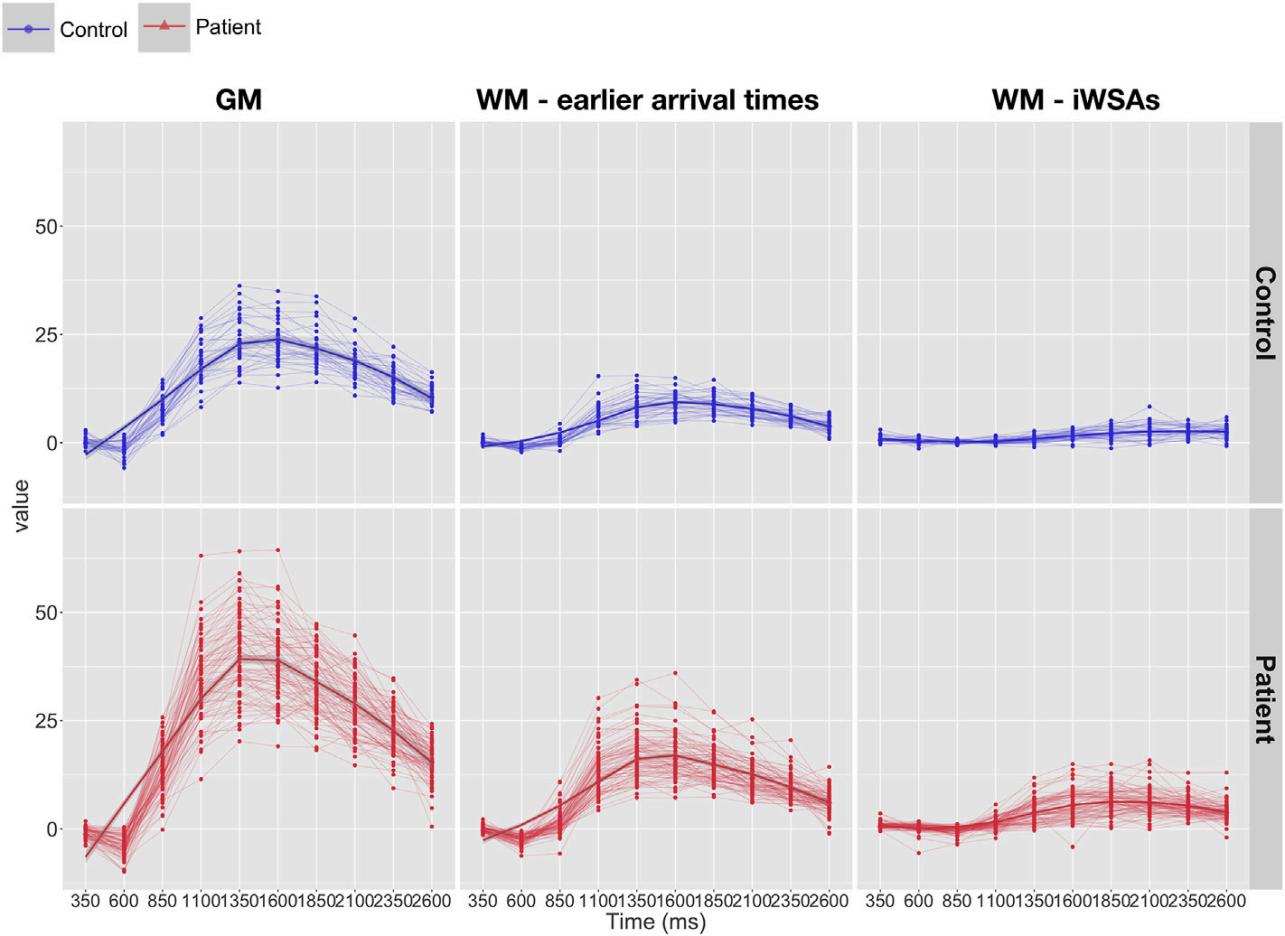
Kinetic curves. Line-plots showing raw kinetic curves (i.e., difference signal at each timepoint) from the multi inflow time sequence across regions of interest in patients (bottom: shown in red) and controls (top: shown in blue). Abbreviations: GM, gray matter; WM, white matter; iWSAs, individual watershed areas.

**FIGURE 5:**
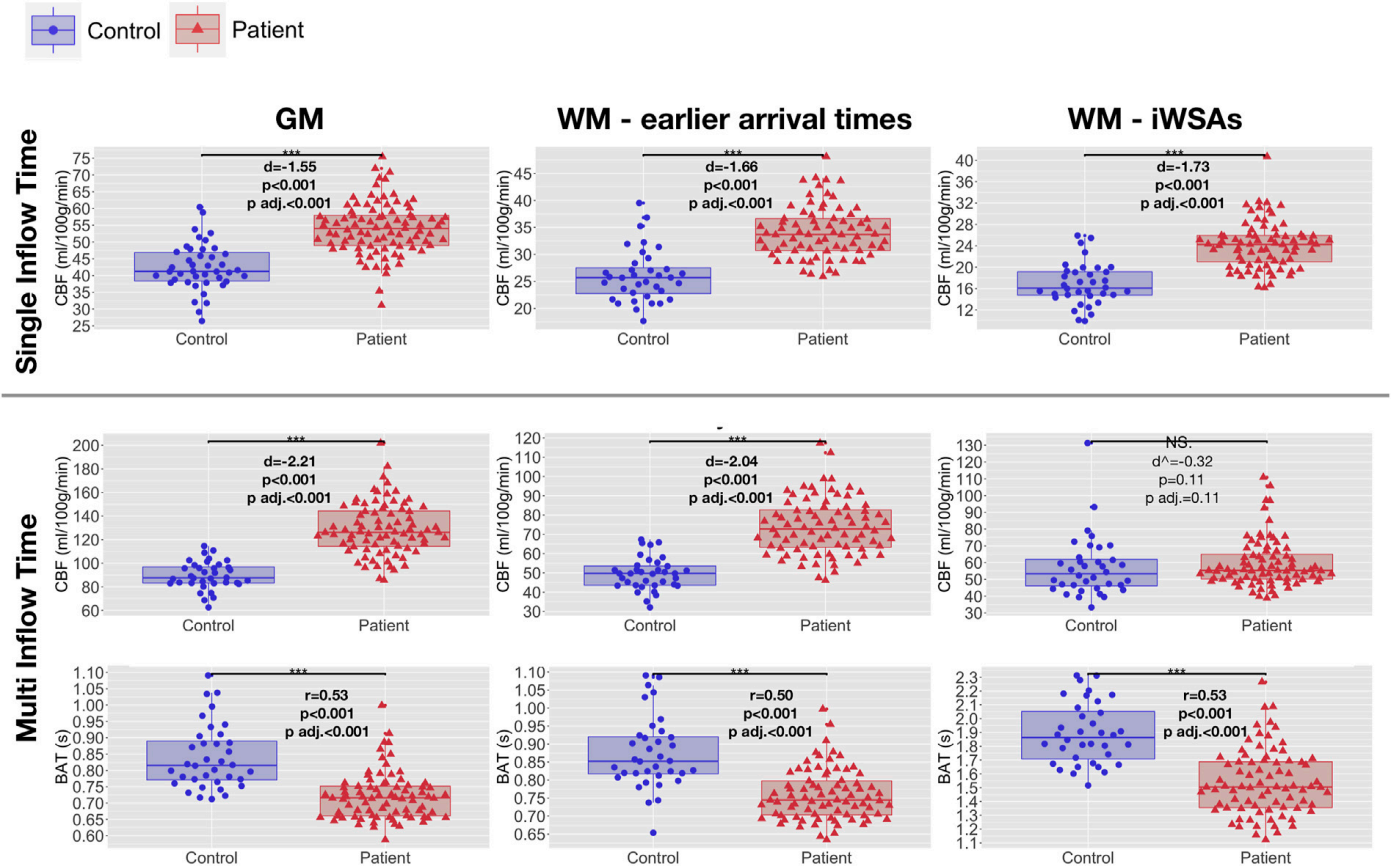
Hemodynamic parameters across regions of interest. Boxplots showing mean cerebral blood flow (CBF) and bolus arrival times (BAT) based on the single- and multi-inflow time sequences (rows) across different regions of interest (ROIs; columns) in patients with sickle cell anemia (shown in red) and healthy controls (shown in blue). Standardized mean differences (d) and probability values from independent t-tests (p) adjusted within parameter types for multiple comparisons using the Benjamini and Hochberg false discovery rate (p adj.) are displayed. ^ between-group differences in iWSA CBF reported with outlying control excluded. Between group differences remain non-significant when the outlier is included (*p* = 0.51). Abbreviations: GM, gray matter; WM, white matter; iWSAs, individual watershed areas.

In both groups, after removal of voxels with low signal to noise ratio, visual inspection of the raw kinetic curves indicated relatively robust multi-TI ASL signal across ROIs, though signal was noticeably lower in controls in the iWSA region (Figure 4). Mean CBF was significantly higher in patients compared to controls across gray matter and white matter with early arrival times, irrespective of sequence (all adjusted *p* < 0.001, Table 1, Figure 5), with large effects sizes. Across iWSAs, there was a discrepancy between sequences (Table 1; Figure 5); estimates based on the single-TI sequence indicated significantly higher mean CBF in patients with SCA with a large effect size (adjusted *p* < 0.001), whereas estimates based on the multi-TI sequence indicated non-significantly higher CBF in patients with SCA (adjusted *p* = 0.20).

Using both sequences, mean BAT was significantly shorter in patients with SCA compared to controls across all ROIs (all adjusted *p* < 0.001, Table 1, Figure 5). Effect sizes were again large. Differences in BAT were on average greatest across iWSAs, where the mean BAT in both groups (Table 1) was longer than the post-labeling delay of the single inflow time sequence (i.e. 1500 ms).

All comparisons remained significant in the same direction when the two patients with large vessel vasculopathy were removed from the sample. There were no significant differences in CBF or BAT across any ROI between controls with and without sickle cell trait (all *p* > 0.05).

### Associations With Arterial Oxygen Content

**FIGURE 6:**
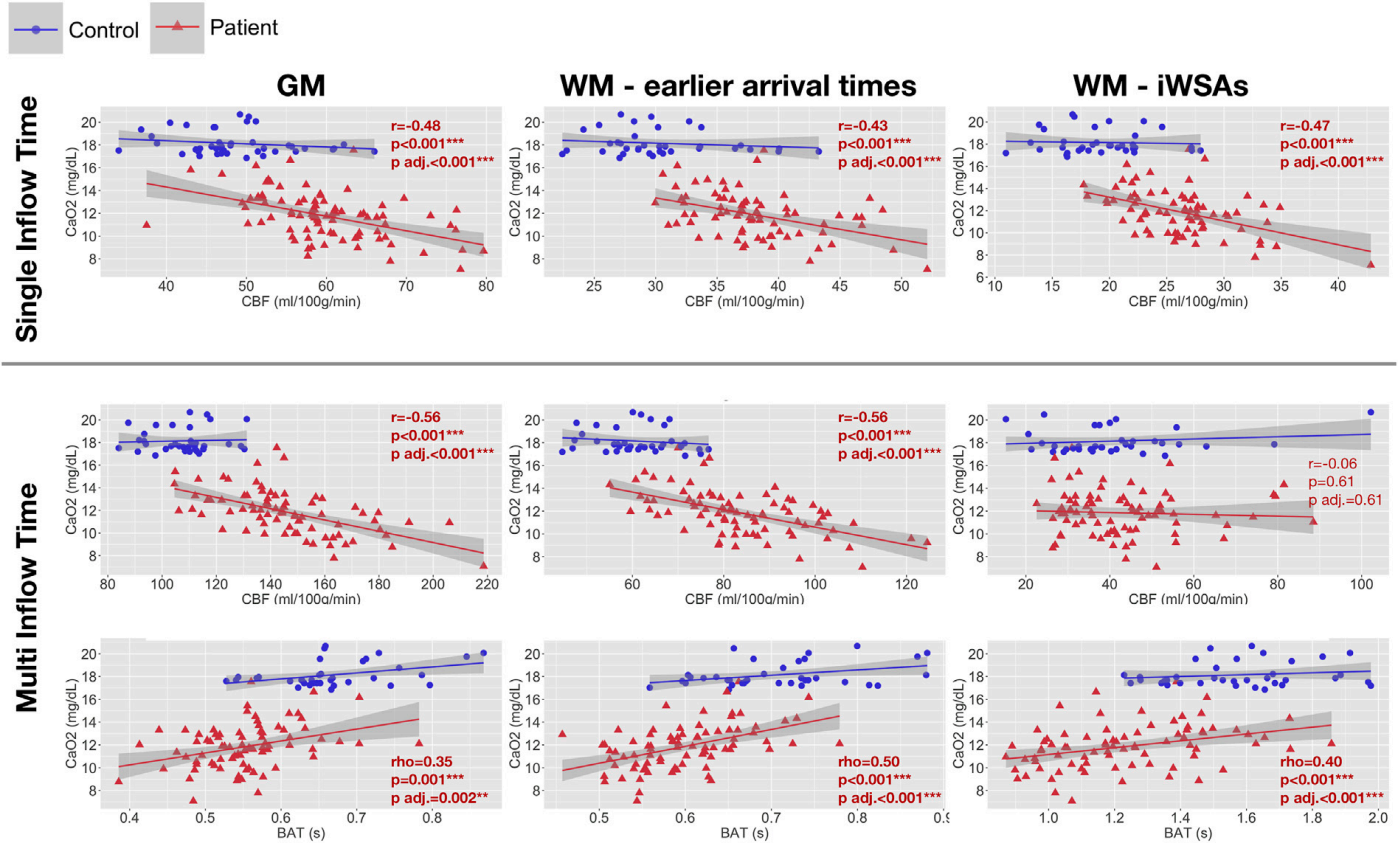
Correlations with arterial oxygen content. Scatterplots showing the relationship between arterial oxygen content and age- and sex-adjusted mean cerebral blood flow (CBF) and bolus arrival time (BAT) based on the single- and multi-inflow time sequences (rows) across different regions of interest (columns) in patients with sickle cell anemia (shown in red). Pearson’s correlation coefficients (r), Spearmans Rho (r) and *p*-values (p) adjusted within parameter types for multiple comparisons using the Benjamini and Hochberg false discovery rate (p adj.) are displayed. Abbreviations: GM, gray matter; WM, white matter; iWSAs, individual watershed areas.

In patients, age- and sex-adjusted mean CBF based on both the single-TI and multi-TI sequence was negatively correlated with arterial oxygen content across gray matter and white matter with early arrival times (all adjusted *p* < 0.001, Figure 6). Across iWSAs, there was a discrepancy between sequences again; arterial oxygen content was negatively correlated with age- and sex-adjusted mean CBF based on the single-TI sequence (adjusted *p* < 0.001, Figure 6), but there was no association with CBF based on the multi-TI sequence (adjusted *p* = 0.47, Figure 6). Age- and sex-adjusted mean BAT was positively correlated with arterial oxygen content across all ROIs irrespective of sequence, (all adjusted *p* < 0.001, Figure 6). Effect sizes were medium to large.

### Associations between parameters across iWSA and lesion burden, microstructural integrity, and cognitive outcome

**FIGURE 7:**
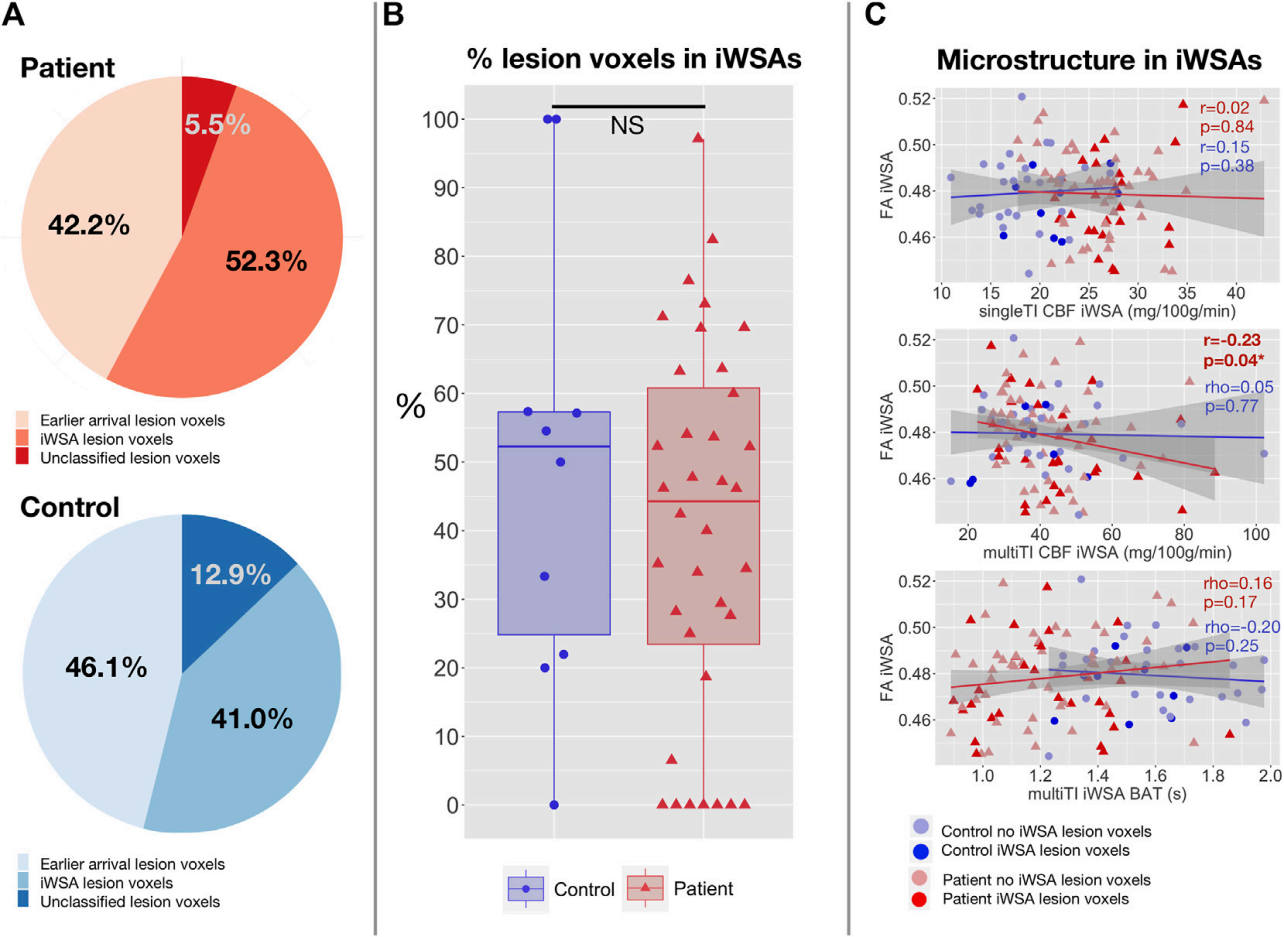
Correlations with lesion burden and microstructural integrity in iWSAs. **(A)** Pie charts showing the proportion of total lesion voxels in white matter with early arrival times, individual watershed areas (iWSAs), and that were unclassified (i.e., excluded due to unreliable signal or eroded to avoid potential partial volume effects) in patients with SCA (top; shown in red) and controls (bottom; shown in blue). **(B)** Boxplot comparing the median proportion of classified lesion voxels in white matter with late arrival times/iWSAs between SCA patients (shown in red) and controls (shown in blue). **(C)** Scatterplots showing the association between age-and sex-adjusted median fractional anisotropy (FA) and age- and sex-mean adjusted cerebral blood flow (CBF) based on the single inflow time sequence (top), CBF based on the multi inflow time sequence (middle) and bolus arrival time (BAT) based on the multi inflow time sequence (bottom) across iWSAs in patients with SCA (shown in red) and controls (shown in blue). Values are Spearman’s correlation coefficients (rho) and *p*-values (p).

There were no significant differences in lesion burden between white matter with early arrival times and iWSAs in patients with SCA or controls (all *p* > 0.05, Figure 7). The median proportion of classified lesion voxels in iWSAs was also not significantly different between groups (*p* = 0.49, Figure 7). Moreover, there were no significant correlations between hemodynamic parameters across iWSA and iWSA lesion burden in patients or controls. However, in patients there were trend-level positive correlations between age- and sex-adjusted iWSA CBF and iWSA lesion burden based on both the single-TI (*rho* = 0.32, *p* = 0.059) and multi-TI (*rho* = 0.32, *p* = 0.069) sequences.

**FIGURE 8:**
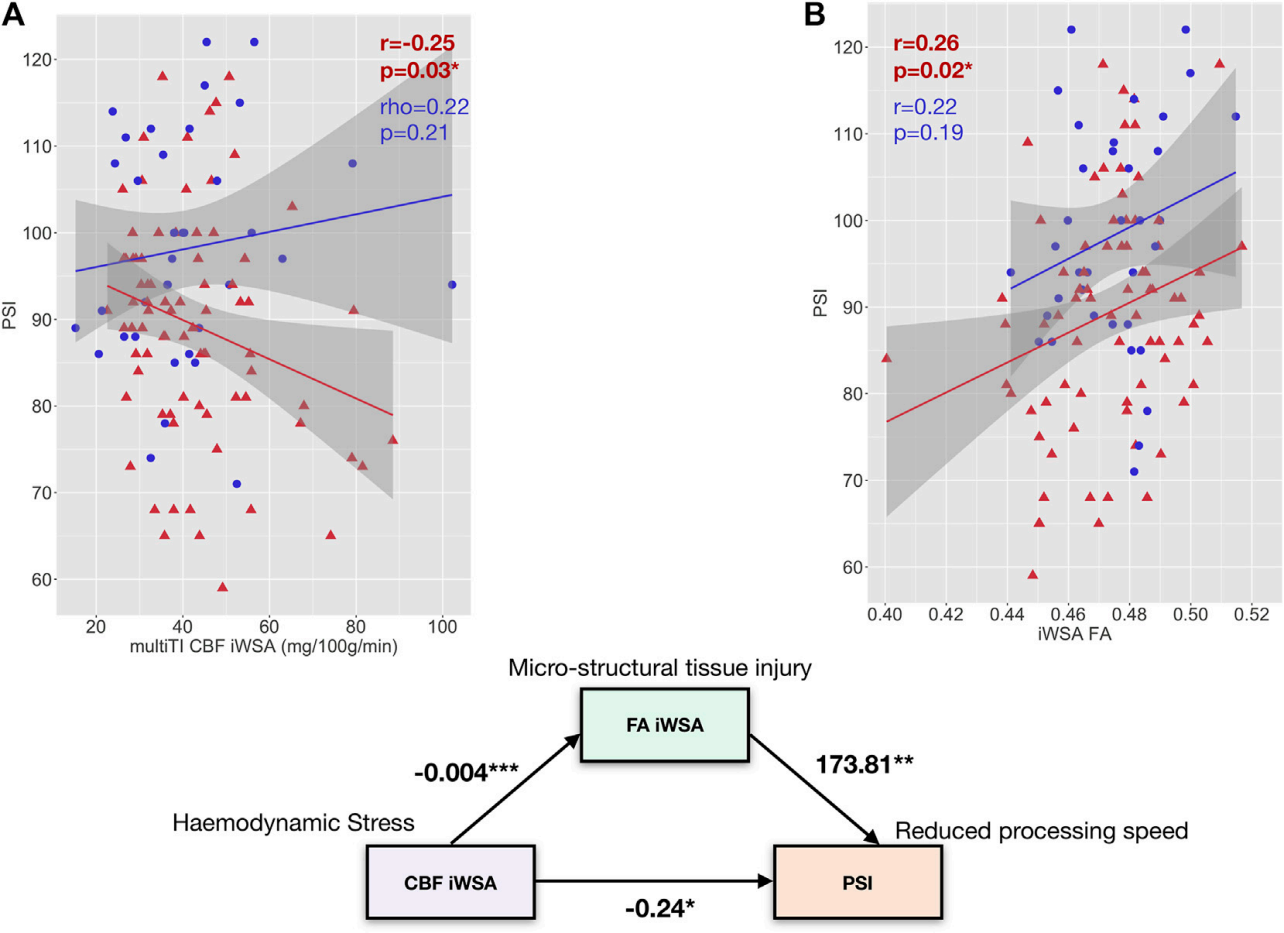
Exploratory Mediation Analysis. **(A)** Scatterplots showing the relationship between PSI and age- and sex-adjusted mean cerebral blood flow (CBF) across individual watershed areas (iWSA) based on the multi-inflow time sequence (left) and fractional anisotropy across iWSA in patients with sickle cell anemia (shown in red) and controls (shown in blue). **(B)**: A path diagram showing the direct effect of mean cerebral blood flow (CBF) across white matter with late arrival times/individual watershed areas (iWSAs) on processing speed index (PSI) along with the indirect effect of CBF on PSI through fractional anisotropy across this region (FA iWSA) in SCD patients. Values are unstandardized regression coefficients. *p < 0.05, **p < 0.01, ***p < 0.001.

There were no associations between age- and sex-adjusted fractional anisotropy across iWSAs and age- and sex-adjusted CBF using the single-TI sequence or BAT using the multi-TI sequence (both *p* > 0.05, Figure 7) in either group. In patients, there was however a small but significant negative correlation in this region between decreases in age- and sex-adjusted fractional anisotropy and increases in age- and sex-adjusted CBF using the multi-TI sequence (*p* = 0.04, Figure 7). Further, in patients with SCA, age- and sex-adjusted mean iWSA CBF based on the multi-TI sequence was negatively correlated with Processing Speed Index (*p* = 0.03, Figure 8), and there was a trend level negative correlation with IQ (*p* = 0.09). Echoing these results, multiple linear regression analysis with Processing Speed Index as the dependent variable found negative effects of CBF based on the multi-TI sequence across iWSAs (*p* = 0.04) and positive effects of fractional anisotropy across this region (*p* = 0.03, Table 2), with small effect sizes. Additional positive effects of arterial oxygen content (*p* = 0.04) and negative effects of male sex (*p* = 0.01) were observed, along with trend-level effects of age (*p* = 0.07), and education deciles (*p* = 0.06). No further associations between hemodynamic parameters across iWSAs and cognitive performance were observed.

**TABLE 2:** Regression Models. Values are unstandardised regression coefficients (b), standardised regression coefficients (β), probability values (p), partial correlation coefficients (r), and 95% confidence intervals (CI) from regression models in patients with sickle cell anemia (SCA). Abbreviations: multiTI, multi inflow time sequence; CBF, cerebral blood flow; iWSA, individual watershed areas; eTIV, estimated total intracranial volume. Significant predictors are shown in bold.

*Predictors*	*b*	β	Outcome: PSI			
				95% CI	p	r
iWSA CBF	−0.21	−0.24		−0.42–0.01	0.04*	−0.24
iWSA FA	158.86	0.24		18.77 298.96	0.03*	0.26
Lesion burden	0.001	0.12		−0.001 0.01	0.28	0.13
Arterial oxygen content	1.54	0.22		0.09 2.99	0.04*	0.25
Age	−0.53	−0.19		−1.10 0.04	0.07	−0.22
Male Sex	−9.79	−0.38		−16.55–3.04	0.01*	−0.33
Chronic Transfusion	−5.59	−0.10		−17.26 6.08	0.34	−0.12
Hydroxycarbamide	−4.42	−0.16		−10.14 1.30	0.13	−0.18
eTIV	0.01	0.13		−0.01 0.04	0.30	0.13
Education Deciles	1.22	0.19		−0.08 2.52	0.06	0.22

Given that CBF across iWSAs was significantly associated with both fractional anisotropy across iWSAs and PSI, an exploratory causal mediation analysis was performed. As Figure 8 illustrates, the regression coefficients between iWSA CBF and iWSA FA, and between iWSA fractional anisotropy and Processing Speed Index, were significant. The effect of CBF across iWSAs on Processing Speed Index was partially mediated by fractional anisotropy across iWSAs. The bootstrapped unstandardised indirect mediation effect was -0.06, (95% CI = -0.16–-0.01, *p* = 0.02), with 27.2% of the effect of iWSA CBF on Processing Speed Index mediated by iWSA fractional anisotropy (Figure 8).

## Discussion

The present study sought to investigate CBF and BAT across gray matter, white matter with early arrival times, and for the first time using ASL, in iWSAs in patients with SCA and controls. In iWSAs, associations between hemodynamic parameters, lesion burden, white matter integrity, and cognitive outcome were also explored. In patients, increases in CBF and reductions in BAT were observed in association with reduced arterial oxygen content across gray matter and white matter with early arrival times using both sequences. Across iWSAs, there was a discrepancy between sequences, with estimates based on the single-TI sequence indicating higher CBF in association with reduced arterial oxygen content in SCA patients, and estimates based on the multi-TI sequence indicating no significant between group differences or associations with arterial oxygen content. Lesion burden was similar between white matter with early arrival times and iWSAs in both patients and controls, and using both sequences, only trend-level associations between iWSA CBF and iWSA lesion burden were observed in patients. Further, using the multi-TI sequence in patients, increased CBF across iWSAs was associated with reduced microstructural tissue integrity and slower processing speed.

### Hemodynamic Parameters

As in prior work (Stotesbury et al., 2022), estimates of CBF based on the multi-TI sequence were increased relative to those based on the single-TI sequence across all regions. This was anticipated, given that the multi-TI sequence captures the flow of labeled water through the large arteries at early arrival times, whereas the single-TI sequence captures flow at a later time, when in healthy parenchyma the labeled water has typically reached the capillary beds. One caveat is that in regions where the blood arrival time is later than the post labeling delay of the single-TI sequence, CBF may be under-estimated.

Despite these differences, in line with prior work, across gray matter and white matter with early arrival times, results based on both ASL sequences indicated higher CBF and shorter BAT in association with reduced arterial oxygen content in patients with SCA. Whilst this was also true for CBF based on the single-TI sequence across iWSAs, the multi-TI sequence indicated no significant differences in CBF between groups in this region, and no association with arterial oxygen content. BAT was again shorter in association with reduced arterial oxygen content in patients across iWSAs, but there was greater variability in patients, and in both groups, average iWSA BAT was longer than the post-labeling delay of the single-TI sequence. This finding suggests that arrival times in iWSAs were too long for the single-TI sequence to adequately capture CBF, and likely explains the discrepancy in results between sequences, underscoring the need for CBF estimates to take BAT into account, as well as the utility of multi-TI ASL for exploration of hemodynamic parameters in these regions.

Taken together, these results support and extend those of prior studies reporting increased gray and white matter CBF and shorter BAT in association with reduced arterial oxygen content in patients with SCA (Bush et al., 2016; Kawadler et al., 2018; Stotesbury et al., 2022). The findings are consistent with the notion that CBF is increased to compensate for reduced blood oxygenation. The findings are however also in agreement with a prior study reporting relatively reduced CBF in regions visually consistent with the deep watershed white matter in patients with SCA (Chai et al., 2019). Here, in a much larger sample and for the first time using ASL, iWSAs were defined physiologically on the basis of individual BAT measurements rather than visually. The resulting iWSA maps fell within the expected regions, demonstrating the feasibility of non-invasive individual iWSA segmentation using multi-TI ASL in patients with SCA. Given that others have reported, albeit visually, that watershed regions continue to exhibit signs of hemodynamic stress following a transfusion (Guilliams et al., 2018), delineation of iWSAs may provide a promising approach in future studies of established and novel treatments, for example in patients who have progressive neurological damage despite adequate transfusion therapy (Scothorn et al., 2002; Hulbert et al., 2011).

In line with the Chai et al. (2019) study, the results from this study similarly suggested relatively reduced CBF in iWSAs, potentially indicating a loss of autoregulatory control in watershed regions. Given that the watershed regions are fed by vessels that are long, narrow, and sparsely distributed, this may be in part due to anatomical constraints, though it is also possible that exhaustion of autoregulatory mechanisms combined with altered capillary/venular flow patterns and functional shunting exacerbate the effect in patients with SCA (Juttukonda et al., 2021; Østergaard et al., 2016; Stotesbury et al., 2019, 2022). The absence of a relationship between CBF and arterial oxygen content observed in this region supports this notion, and may suggest oxygen supply-demand mismatch as a result.

### Associations With Lesions

However, contrary to expectations and prior work, lesion burden was not significantly greater in iWSAs compared to white matter with earlier arrival times in patients with SCA or controls. The discrepancy with prior studies in terms of lesion distribution may relate to our use of a high-resolution FLAIR sequence, which likely increased lesion detectability. Increased lesion detectability is consistent with the relatively high proportion of controls detected with lesions, as seen in other high resolution MRI studies of patients with SCA (Chai et al., 2019). While prior studies used manual delineation of lesions, we also used a semi-automated pipeline, which ensured that objective intensity criteria were applied. Use of a minimum length requirement for lesions in prior studies, and prior heat-map methods involving manual delineation of lesions with exclusion of lesions in less commonly affected regions, may also have played a role (Fields et al., 2018; Ford et al., 2018).

The absence of a spatial relationship between lesions and iWSAs suggests that ischemic tissue injury is not solely explicable by an exacerbation of the watershed effect in patients with SCA, though this may play a role in some patients, particularly during acute illness and/or in patients with severe flow-limiting vasculopathy. Prior work has demonstrated acute ischemic lesions on diffusion weighted imaging in watershed regions in both acutely ill and steady-state patients with SCA, with only some of these transitioning to lesions observable on FLAIR (Dowling et al., 2012; Quinn et al., 2013). Acute drops in oxygen delivery during illness, in combination with inefficient oxygen extraction secondary to hyperemic flow and/or altered capillary/venular flow patterns, may plausibly play a role in some patients.

It is notable that whilst the majority of patients with SCA and controls with lesions did have at least some lesion burden in iWSAs, there was substantial heterogeneity, along with a subset of patients (*n* = 7, 20% of patients with lesions) with lesion voxels in white matter regions with early arrival times only. It is unclear whether other mechanisms are at play altogether in this subset of patients (Dowling et al., 2009), such as embolization related to shunting through the heart or lungs (Dowling et al., 2010, 2017; Dowling and Ikemba, 2011), or changes related to posterior reversible encephalopathy syndrome. There has been discussion of potential “hyper-hemolytic—endothelial dysfunction” and “hyper-viscose—vaso-occlusive” sub-phenotypes of SCA (Connes et al., 2016; Hebbel, 2011; Kato et al., 2007, 2017). Much more work is however required to understand the etiology of lesions in all patients as well as in controls. Given that lesions are not detected at the time they occur, longitudinal multi-modal studies investigating potential hematologic and hemodynamic factors involved in transition from acute to established lesions, along with potential downstream effects on tissue structure and function, are likely to be most useful.

Further challenging the notion that ischemic tissue injury is primarily a result of an exacerbation of the watershed effect in SCA, only trend-level associations between lesion burden in iWSAs and iWSA CBF were observed, and the associations were positive rather than negative. The “functional shunting” model offers a possible explanation for this counterintuitive finding (Østergaard et al., 2016). According to this model, during rest in healthy populations, capillary flow is heterogenous, limiting net oxygen extraction, referred to as “functional shunting”. When CBF increases in relation to cortical activation, flow homogenises, facilitating more efficient oxygen unloading. Within this model, capillary dysfunction that disrupts capillary flow patterns, increasing their heterogeneity, or disrupting their homogenization, is postulated as a potential mechanism that may decrease oxygen extraction efficacy, leading to ischemic injury even in the absence of flow-limiting pathology or hypoxic exposure, as is observed in other populations with cerebral small vessel disease (Østergaard et al., 2016). Importantly, the model proposes that as capillary dysfunction worsens, increases in CBF may not only no longer maintain tissue oxygen tension, but also paradoxically reduce it, by exacerbating functional shunting (Østergaard et al., 2016). Under such conditions where capillary transit time heterogeneity cannot be reduced, the model proposes that only the suppression of CBF can reduce functional shunting.

### Associations With Microstructural Integrity and Cognitive Outcome

Consistent with the notion that increased flow may be non-nutritive, higher CBF across iWSAs was associated with reduced fractional anisotropy and slower processing speed in patients with SCA. These findings were echoed in a regression model where independent effects of both increased iWSA CBF and reduced iWSA FA on processing speed were observed. These findings support and extend those of prior DTI studies reporting tissue integrity reductions that appear to be functionally significant in patients with SCA (Kawadler et al., 2015; Stotesbury et al., 2018b; Jacob et al., 2020; Chai et al., 2021). They are also in line with one recent study reporting an association between increased OEF and increased mean diffusivity in SCA patients (Wang et al., 2021). The study also reported, albeit visually, that regions exhibiting hemodynamic stress, reflected by increases in OEF, encompassed watershed regions of elevated mean diffusivity and reduced fractional anisotropy, which the authors noted extended beyond the locations of lesions (Wang et al., 2021).

Here, in an exploratory mediation analysis, iWSA fractional anisotropy was found to partially mediate the relationship between higher iWSA CBF and slower processing speed, indicating potential downstream effects of regional hemodynamic stress on tissue integrity, in turn leading to slower processing speed indirectly. The remaining direct link observed is interesting, and aligns with a relatively recent study reporting improvement in executive function near as opposed to far away from transfusion, potentially consistent with a direct, but temporary, effect of increased tissue oxygen delivery, perhaps via amelioration of fatigue (Hood et al., 2019).

### Limitations

Use of single-TI and multi-TI ASL to measure hemodynamic parameters in patients with SCA is limited by several factors, all of which have been described in depth previously (Bush A. et al., 2018; Stotesbury et al., 2022). Of particular note for the design of this study, it is important that inflow times are optimised for the regions of interest. Examination of the raw kinetic curves indicated reasonable signal across all examined regions. However, signal was notably lower across both white matter regions, particularly in iWSAs in controls, which may have reduced the accuracy of estimates in this region. We cannot exclude the possibility that had we included further images at longer TIs, further CBF signal may have been detected in controls in this region. However, the extended inflow times required for this would have resulted in significant T1 recovery of the inverted blood-water, which would further reduce the signal to noise ratio. We attempted to correct for reduced signal to noise ratio by our inclusion of a noise removal step, which ensured that voxels in which the signal was lower than the mean plus one standard deviation of background noise were removed from the analysis. Inclusion of further images would also have increased the scan time and motion susceptibility.

Our study was also limited by our lack of hemoglobin and hematocrit measures in controls. Ethics approval committees in the United Kingdom will frequently not allow invasive procedures in healthy children (i.e., blood draws) unless a direct benefit to the children themselves can be demonstrated. People with sickle cell trait have hemoglobin levels within the normal range and we do not consider that the lack of direct measurement would alter our results although it should be possible to confirm this using data obtained in other countries where blood draws are part of routine screening in healthy children. We also did not have a direct measure of socioeconomic status, though self-report measures are also subject to limitations and our postcode-based index did show some sensitivity to processing speed in our regression model.

## Conclusion

In summary, our findings demonstrate the feasibility of multi-TI ASL for objective delineation of iWSAs, which have previously been identified as at risk of tissue injury in patients with SCA. Further, our findings highlight the utility of this technique for identification of hemodynamic stress in iWSAs that is associated with reduced microstructural tissue integrity and slower processing speed. Future studies should explore the predictive validity of regional multi-TI parameters longitudinally.

## Data Availability

The data supporting the conclusion of this article will be made available by the authors, without undue reservation.

## References

[B1] Alsop D. C. Detre J. A. Golay X. Günther M. Hendrikse J. Hernandez-Garcia L. (2015). Recommended Implementation of Arterial Spin-Labeled Perfusion MRI for Clinical Applications: A Consensus of the ISMRM Perfusion Study Group and the European Consortium for ASL in Dementia. Magn. Reson. Med. 73, 102–116. 10.1002/mrm.25197 24715426 PMC4190138

[B2] Aygun B. Odame I. (2012). A Global Perspective on Sickle Cell Disease. Pediatr. Blood Cancer 59, 386–390. 10.1002/pbc.24175 22535620

[B3] Behpour A. M. Shah P. S. Mikulis D. J. Kassner A. (2013). Cerebral Blood Flow Abnormalities in Children with Sickle Cell Disease: a Systematic Review. Pediatr. Neurol. 48, 188–199. 10.1016/j.pediatrneurol.2012.12.004 23419469

[B4] Benjamini Y. Hochberg Y. (1995). Controlling the False Discovery Rate: A Practical and Powerful Approach to Multiple Testing. J. R. Stat. Soc. Ser. B (Methodological) 57, 289–300. 10.1111/j.2517-6161.1995.tb02031.x

[B5] Brousse V. Pondarre C. Kossorotoff M. Arnaud C. Kamdem A. Montalembert M. d. (2021). Brain Injury Pathophysiology Study by a Multimodal Approach in Children with Sickle Cell Anemia with No Intra or Extra Cranial Arteriopathy. haematol. 10.3324/haematol.2020.278226 PMC8968884 33882639

[B6] Bush A. Chai Y. Choi S. Y. Vaclavu L. Holland S. Nederveen A. (2018a). Pseudo Continuous Arterial Spin Labeling Quantification in Anemic Subjects with Hyperemic Cerebral Blood Flow. Magn. Reson. Imaging 47, 137–146. 10.1016/j.mri.2017.12.011 29229306 PMC5834316

[B7] Bush A. M. Borzage M. T. Choi S. Václavů L. Tamrazi B. Nederveen A. J. (2016). Determinants of Resting Cerebral Blood Flow in Sickle Cell Disease. Am. J. Hematol. 91, 912–917. 10.1002/ajh.24441 27263497 PMC4987198

[B8] Bush A. M. Coates T. D. Wood J. C. (2018b). Diminished Cerebral Oxygen Extraction and Metabolic Rate in Sickle Cell Disease Using T2 Relaxation under Spin Tagging MRI. Magn. Reson. Med. 80, 294–303. 10.1002/mrm.27015 29194727 PMC5876140

[B9] Buxton R. B. Frank L. R. Wong E. C. Siewert B. Warach S. Edelman R. R. (1998). A General Kinetic Model for Quantitative Perfusion Imaging with Arterial Spin Labeling. Magn. Reson. Med. 40, 383–396. 10.1002/mrm.1910400308 9727941

[B10] Castro O. L. Haddy T. B. Rana S. R. (1987). Age- and Sex-Related Blood Cell Values in Healthy Black Americans. Public Health Rep. 102, 232–237. 3104982 PMC1477821

[B11] Chai Y. Bush A. M. Coloigner J. Nederveen A. J. Tamrazi B. Vu C. (2019). White Matter Has Impaired Resting Oxygen Delivery in Sickle Cell Patients. Am. J. Hematol. 94, 467–474. 10.1002/ajh.25423 30697803 PMC6874897

[B12] Chai Y. Ji C. Coloigner J. Choi S. Balderrama M. Vu C. (2021). Tract‐specific Analysis and Neurocognitive Functioning in Sickle Cell Patients without History of Overt Stroke. Brain Behav. 11, e01978. 10.1002/brb3.1978 33434353 PMC7994688

[B13] Chaturvedi S. Ghafuri D. L. Jordan N. Kassim A. Rodeghier M. DeBaun M. R. (2018). Clustering of End-Organ Disease and Earlier Mortality in Adults with Sickle Cell Disease: A Retrospective-Prospective Cohort Study. Am. J. Hematol. 93, 1153–1160. 10.1002/ajh.25202 29981283

[B14] Cohen J. (1988). Statistical Power Analysis for the Behavioral Sciences. 2nd Edn. New York, NY: Acad. Press.

[B15] Connes P. Alexy T. Detterich J. Romana M. Hardy-Dessources M.-D. Ballas S. K. (2016). The Role of Blood Rheology in Sickle Cell Disease. Blood Rev. 30, 111–118. 10.1016/j.blre.2015.08.005 26341565 PMC6447059

[B16] Dowling M. M. Ikemba C. M. (2011). Intracardiac Shunting and Stroke in Children: a Systematic Review. J. Child. Neurol. 26, 72–82. 10.1177/0883073810383913 21212453 PMC5642963

[B17] Dowling M. M. Lee N. Quinn C. T. Rogers Z. R. Boger D. Ahmad N. (2010). Prevalence of Intracardiac Shunting in Children with Sickle Cell Disease and Stroke. J. Pediatr. 156, 645–650. 10.1016/j.jpeds.2009.10.012 20022343 PMC4250927

[B18] Dowling M. M. Quinn C. T. Plumb P. Rogers Z. R. Rollins N. K. Koral K. (2012). Acute Silent Cerebral Ischemia and Infarction during Acute Anemia in Children with and without Sickle Cell Disease. Blood 120, 3891–3897. 10.1182/blood-2012-01-406314 22948048 PMC3496951

[B19] Dowling M. M. Quinn C. T. Ramaciotti C. Kanter J. Osunkwo I. Inusa B. (2017). Increased Prevalence of Potential Right-To-Left Shunting in Children with Sickle Cell Anaemia and Stroke. Br. J. Haematol. 176, 300–308. 10.1111/bjh.14391 27766637 PMC5239723

[B20] Dowling M. M. Quinn C. T. Rogers Z. R. Journeycake J. M. (2009). Stroke in Sickle Cell Anemia: Alternative Etiologies. Pediatr. Neurol. 41, 124–126. 10.1016/j.pediatrneurol.2009.02.011 19589461 PMC2740936

[B21] Ellison A. M. Shaw K. (2007). Management of Vasoocclusive Pain Events in Sickle Cell Disease. Pediatr. Emerg. Care 23, 832–841. 10.1097/PEC.0b013e31815a05e2 18007218

[B22] Fields M. E. Guilliams K. P. Ragan D. Eldeniz C. Binkley M. Hulbert M. L. (2015). Elevations in MR Measurements of Whole Brain and Regional Cerebral Blood Flow and Oxygen Extraction Fraction Suggest Cerebral Metabolic Stress in Children with Sickle Cell Disease Unaffected by Overt Stroke. Blood 126, 69. 10.1182/blood.v126.23.69.69 25987655

[B23] Fields M. E. Guilliams K. P. Ragan D. K. Binkley M. M. Eldeniz C. Chen Y. (2018). Regional Oxygen Extraction Predicts Border Zone Vulnerability to Stroke in Sickle Cell Disease. Neurology 90, e1134–e1142. 10.1212/WNL.0000000000005194 29500287 PMC5880632

[B24] Ford A. L. Ragan D. K. Fellah S. Binkley M. M. Fields M. E. Guilliams K. P. (2018). Silent Infarcts in Sickle Cell Disease Occur in the Border Zone Region and Are Associated with Low Cerebral Blood Flow. Blood 132, 1714–1723. 10.1182/blood-2018-04-841247 30061156 PMC6194388

[B25] Gardner K. Douiri A. Drasar E. Allman M. Mwirigi A. Awogbade M. (2016). Survival in Adults with Sickle Cell Disease in a High-Income Setting. Blood 128, 1436–1438. 10.1182/blood-2016-05-716910 27439910

[B26] Guilliams K. P. Fields M. E. Ragan D. K. Eldeniz C. Binkley M. M. Chen Y. (2018). Red Cell Exchange Transfusions Lower Cerebral Blood Flow and Oxygen Extraction Fraction in Pediatric Sickle Cell Anemia. Blood 131, 1012–1021. 10.1182/blood-2017-06-789842 29255068 PMC5833262

[B27] Hales P. W. Kirkham F. J. Clark C. A. (2016). A General Model to Calculate the Spin-Lattice (T1) Relaxation Time of Blood, Accounting for Haematocrit, Oxygen Saturation and Magnetic Field Strength. J. Cereb. Blood Flow Metab. 36, 370–374. 10.1177/0271678X15605856 26661147 PMC4759664

[B28] Hebbel R. P. (2011). Reconstructing Sickle Cell Disease: A Data-Based Analysis of the "hyperhemolysis Paradigm" for Pulmonary Hypertension from the Perspective of Evidence-Based Medicine. Am. J. Hematol. 86, 123–154. 10.1002/ajh.21952 21264896

[B29] Helton K. J. Paydar A. Glass J. Weirich E. M. Hankins J. Li C.-S. (2009). Arterial Spin-Labeled Perfusion Combined with Segmentation Techniques to Evaluate Cerebral Blood Flow in white and gray Matter of Children with Sickle Cell Anemia. Pediatr. Blood Cancer 52, 85–91. 10.1002/pbc.21745 18937311 PMC4480678

[B30] Hood A. M. King A. A. Fields M. E. Ford A. L. Guilliams K. P. Hulbert M. L. (2019). Higher Executive Abilities Following a Blood Transfusion in Children and Young Adults with Sickle Cell Disease. Pediatr. Blood Cancer 66, e27899. 10.1002/pbc.27899 31267645 PMC6707832

[B31] Howard J. Slee A. E. Skene S. Inusa B. Kawadler J. Downes M. (2018). Overnight Auto-Adjusting Continuous Airway Pressure + Standard Care Compared with Standard Care Alone in the Prevention of Morbidity in Sickle Cell Disease Phase II (POMS2b): Study Protocol for a Randomised Controlled Trial. Trials 19, 55. 10.1186/s13063-017-2419-0 29357947 PMC5778753

[B32] Hulbert M. L. McKinstry R. C. Lacey J. L. Moran C. J. Panepinto J. A. Thompson A. A. (2011). Silent Cerebral Infarcts Occur Despite Regular Blood Transfusion Therapy after First Strokes in Children with Sickle Cell Disease. Blood 117, 772–779. 10.1182/blood-2010-01-261123 20940417 PMC3035071

[B33] Jacob M. Stotesbury H. Kawadler J. M. Lapadaire W. Saunders D. E. Sangeda R. Z. (2020). White Matter Integrity in Tanzanian Children with Sickle Cell Anemia. Stroke 51, 1166–1173. 10.1161/STROKEAHA.119.027097 32138633

[B34] Jordan L. C. DeBaun M. R. (2018). Cerebral Hemodynamic Assessment and Neuroimaging across the Lifespan in Sickle Cell Disease. J. Cereb. Blood Flow Metab. 38, 1438–1448. 10.1177/0271678X17701763 28417646 PMC6125971

[B35] Jordan L. C. Gindville M. C. Scott A. O. Juttukonda M. R. Strother M. K. Kassim A. A. (2016). Non-invasive Imaging of Oxygen Extraction Fraction in Adults with Sickle Cell Anaemia. Brain 139, 738–750. 10.1093/brain/awv397 26823369 PMC5014126

[B36] Juttukonda M. R. Donahue M. J. Davis L. T. Gindville M. C. Lee C. A. Patel N. J. (2019). Preliminary Evidence for Cerebral Capillary Shunting in Adults with Sickle Cell Anemia. J. Cereb. Blood Flow Metab. 39, 1099–1110. 10.1177/0271678X17746808 29260615 PMC6547194

[B37] Juttukonda M. R. Donahue M. J. Waddle S. L. Davis L. T. Lee C. A. Patel N. J. (2021). Reduced Oxygen Extraction Efficiency in Sickle Cell Anemia Patients with Evidence of Cerebral Capillary Shunting. J. Cereb. Blood Flow Metab. 41, 546–560. 10.1177/0271678X20913123 32281458 PMC7922746

[B38] Juttukonda M. R. Jordan L. C. Gindville M. C. Davis L. T. Watchmaker J. M. Pruthi S. (2017). Cerebral Hemodynamics and Pseudo-continuous Arterial Spin Labeling Considerations in Adults with Sickle Cell Anemia. NMR Biomed. 30, e3681. 10.1002/nbm.3681 PMC5351809 28052565

[B39] Kaczmarz S. Göttler J. Petr J. Hansen M. B. Mouridsen K. Zimmer C. (2021). Hemodynamic Impairments within Individual Watershed Areas in Asymptomatic Carotid Artery Stenosis by Multimodal MRI. J. Cereb. Blood Flow Metab. 41, 380–396. 10.1177/0271678X20912364 32237952 PMC7812517

[B40] Kato G. J. Gladwin M. T. Steinberg M. H. (2007). Deconstructing Sickle Cell Disease: Reappraisal of the Role of Hemolysis in the Development of Clinical Subphenotypes. Blood Rev. 21, 37–47. 10.1016/j.blre.2006.07.001 17084951 PMC2048670

[B41] Kato G. J. Steinberg M. H. Gladwin M. T. (2017). Intravascular Hemolysis and the Pathophysiology of Sickle Cell Disease. J. Clin. Invest. 127, 750–760. 10.1172/JCI89741 28248201 PMC5330745

[B42] Kawadler J. M. Hales P. W. Barker S. Cox T. C. S. Kirkham F. J. Clark C. A. (2018). Cerebral Perfusion Characteristics Show Differences in Younger versus Older Children with Sickle Cell Anaemia: Results from a Multiple-Inflow-Time Arterial Spin Labelling Study. NMR Biomed. 31, e3915. 10.1002/nbm.3915 29601112

[B43] Kawadler J. M. Kirkham F. J. Clayden J. D. Hollocks M. J. Seymour E. L. Edey R. (2015). White Matter Damage Relates to Oxygen Saturation in Children with Sickle Cell Anemia without Silent Cerebral Infarcts. Stroke 46, 1793–1799. 10.1161/STROKEAHA.115.008721 25967572

[B44] Kellner E. Dhital B. Kiselev V. G. Reisert M. (2016). Gibbs-ringing Artifact Removal Based on Local Subvoxel-Shifts. Magn. Reson. Med. 76, 1574–1581. 10.1002/mrm.26054 26745823

[B45] Kim J. A. Leung J. Lerch J. P. Kassner A. (2016). Reduced Cerebrovascular reserve Is Regionally Associated with Cortical Thickness Reductions in Children with Sickle Cell Disease. Brain Res. 1642, 263–269. 10.1016/j.brainres.2016.03.041 27026656

[B46] Kirkham F. J. Calamante F. Bynevelt M. Gadian D. G. Evans J. P. M. Cox T. C. (2001). Perfusion Magnetic Resonance Abnormalities in Patients with Sickle Cell Disease. Ann. Neurol. 49, 477–485. 10.1002/ana.97 11310625

[B47] Kosinski P. D. Croal P. L. Leung J. Williams S. Odame I. Hare G. M. T. (2017). The Severity of Anaemia Depletes Cerebrovascular Dilatory reserve in Children with Sickle Cell Disease: a Quantitative Magnetic Resonance Imaging Study. Br. J. Haematol. 176, 280–287. 10.1111/bjh.14424 27905100

[B48] Luh W.-M. Wong E. C. Bandettini P. A. Hyde J. S. (1999). QUIPSS II with Thin-Slice TI1 Periodic Saturation: A Method for Improving Accuracy of Quantitative Perfusion Imaging Using Pulsed Arterial Spin Labeling. Magn. Reson. Med. 41, 1246–1254. 10.1002/(sici)1522-2594(199906)41:6<1246::aid-mrm22>3.0.co;2-n 10371458

[B49] McCrimmon A. W. Smith A. D. (2013). Review of the Wechsler Abbreviated Scale of Intelligence, Second Edition (WASI-II). J. Psychoeducational Assess. 31, 337–341. 10.1177/0734282912467756

[B50] Milner P. F. (1974). Oxygen Transport in Sickle Cell Anemia. Arch. Intern. Med. 133, 565–572. 10.1001/archinte.1974.00320160059006 4594394

[B51] Nath K. A. Katusic Z. S. Gladwin M. T. (2004). The Perfusion Paradox and Vascular Instability in Sickle Cell Disease. Microcirculation 11, 179–193. 10.1080/10739680490278592 15280091

[B52] Needleman J. P. Setty B. N. Varlotta L. Dampier C. Allen J. L. (1999). Measurement of Hemoglobin Saturation by Oxygen in Children and Adolescents with Sickle Cell Disease. Pediatr. Pulmonol. 28, 423–428. 10.1002/(sici)1099-0496(199912)28:6<423::aid-ppul7>3.0.co;2-c 10587417

[B53] Nur E. Kim Y.-S. Truijen J. van Beers E. J. Davis S. C. A. T. Brandjes D. P. (2009). Cerebrovascular reserve Capacity Is Impaired in Patients with Sickle Cell Disease. Blood 114, 3473–3478. 10.1182/blood-2009-05-223859 19700663

[B54] Østergaard L. Engedal T. S. Moreton F. Hansen M. B. Wardlaw J. M. Dalkara T. (2016). Cerebral Small Vessel Disease: Capillary Pathways to Stroke and Cognitive Decline. J. Cereb. Blood Flow Metab. 36, 302–325. 10.1177/0271678X15606723 26661176 PMC4759673

[B55] Prohovnik I. Hurlet-Jensen A. Adams R. De Vivo D. Pavlakis S. G. (2009). Hemodynamic Etiology of Elevated Flow Velocity and Stroke in Sickle-Cell Disease. J. Cereb. Blood Flow Metab. 29, 803–810. 10.1038/jcbfm.2009.6 19209182

[B56] Prussien K. V. Jordan L. C. DeBaun M. R. Compas B. E. (2019). Cognitive Function in Sickle Cell Disease across Domains, Cerebral Infarct Status, and the Lifespan: A Meta-Analysis. J. Pediatr. Psychol. 44, 948–958. 10.1093/jpepsy/jsz031 31050352 PMC6706005

[B57] Quinn C. T. McKinstry R. C. Dowling M. M. Ball W. S. Kraut M. A. Casella J. F. (2013). Acute Silent Cerebral Ischemic Events in Children with Sickle Cell Anemia. JAMA Neurol. 70, 58–65. 10.1001/jamaneurol.2013.576 23108767 PMC3677221

[B58] Rosen C. L. Debaun M. R. Strunk R. C. Redline S. Seicean S. Craven D. I. (2014). Obstructive Sleep Apnea and Sickle Cell Anemia. Pediatrics 134, 273–281. 10.1542/peds.2013-4223 25022740 PMC4187233

[B59] Scothorn D. J. Price C. Schwartz D. Terrill C. Buchanan G. R. Shurney W. (2002). Risk of Recurrent Stroke in Children with Sickle Cell Disease Receiving Blood Transfusion Therapy for at Least Five Years after Initial Stroke. J. Pediatr. 140, 348–354. 10.1067/mpd.2002.122498 11953734

[B60] Smith S. M. Jenkinson M. Woolrich M. W. Beckmann C. F. Behrens T. E. J. Johansen-Berg H. (2004). Advances in Functional and Structural MR Image Analysis and Implementation as FSL. Neuroimage 23, S208–S219. 10.1016/j.neuroimage.2004.07.051 15501092

[B61] Stotesbury H. Hales P. W. Kirkham F. J. (2018a). The Promise of Noninvasive Cerebral Hemodynamic Assessment in Sickle Cell Anemia. Neurology 90, 585–586. 10.1212/WNL.0000000000005236 29500289

[B62] Stotesbury H. Hales P. W. Koelbel M. Hood A. M. Kawadler J. M. Saunders D. E. (2022). Venous Cerebral Blood Flow Quantification and Cognition in Patients with Sickle Cell Anemia. J. Cereb. Blood Flow Metab., 0271678X2110723. 10.1177/0271678X211072391 PMC9121533 34986673

[B63] Stotesbury H. Kawadler J. M. Hales P. W. Saunders D. E. Clark C. A. Kirkham F. J. (2019). Vascular Instability and Neurological Morbidity in Sickle Cell Disease: An Integrative Framework. Front. Neurol. 10, 871. 10.3389/fneur.2019.00871 31474929 PMC6705232

[B64] Stotesbury H. Kawadler J. M. Saunders D. E. Kirkham F. J. (2021). MRI Detection of Brain Abnormality in Sickle Cell Disease. Expert Rev. Hematol. 14, 473–491. 10.1080/17474086.2021.1893687 33612034 PMC8315209

[B65] Stotesbury H. Kirkham F. J. Kölbel M. Balfour P. Clayden J. D. Sahota S. (2018b). White Matter Integrity and Processing Speed in Sickle Cell Anemia. Neurology 90, e2042–e2050. 10.1212/WNL.0000000000005644 29752305 PMC5993179

[B66] Tingley D. Yamamoto T. Hirose K. Keele L. Imai K. (2014). mediation:RPackage for Causal Mediation Analysis. J. Stat. Soft. 59. 10.18637/jss.v059.i05

[B67] Tournier J.-D. Smith R. Raffelt D. Tabbara R. Dhollander T. Pietsch M. (2019). MRtrix3: A Fast, Flexible and Open Software Framework for Medical Image Processing and Visualisation. Neuroimage 202, 116137. 10.1016/j.neuroimage.2019.116137 31473352

[B68] Václavů L. Meynart B. N. Mutsaerts H. J. M. M. Petersen E. T. Majoie C. B. L. M. VanBavel E. T. (2019). Hemodynamic Provocation with Acetazolamide Shows Impaired Cerebrovascular reserve in Adults with Sickle Cell Disease. Haematologica 104, 690–699. 10.3324/haematol.2018.206094 30523051 PMC6442969

[B69] Vaclavu L. Petersen E. T. VanBavel E. T. Majoie C. B. Nederveen A. J. Biemond B. J. (2018). Reduced Cerebral Metabolic Rate of Oxygen in Adults with Sickle Cell Disease. Blood 132, 11. 10.1182/BLOOD-2018-99-116194

[B70] van den Tweel X. W. Nederveen A. J. Majoie C. B. L. M. van der Lee J. H. Wagener-Schimmel L. Van Walderveen M. A. A. (2009). Cerebral Blood Flow Measurement in Children with Sickle Cell Disease Using Continuous Arterial Spin Labeling at 3.0-Tesla MRI. Stroke 40, 795–800. 10.1161/STROKEAHA.108.523308 19150876

[B71] van der Land V. Hijmans C. T. de Ruiter M. Mutsaerts H. J. M. M. Cnossen M. H. Engelen M. (2015a). Volume of white Matter Hyperintensities Is an Independent Predictor of Intelligence Quotient and Processing Speed in Children with Sickle Cell Disease. Br. J. Haematol. 168, 553–556. 10.1111/bjh.13179 25303108

[B72] van der Land V. Zwanenburg J. J. M. Fijnvandraat K. Biemond B. J. Hendrikse J. Mutsaerts H. J. M. M. (2015b). Cerebral Lesions on 7 Tesla MRI in Patients with Sickle Cell Anemia. Cerebrovasc. Dis. 39, 181–189. 10.1159/000373917 25765995

[B73] Vu C. Bush A. Choi S. Borzage M. Miao X. Nederveen A. J. (2021). Reduced Global Cerebral Oxygen Metabolic Rate in Sickle Cell Disease and Chronic Anemias. Am. J Hematol 96, 901–913. 10.1002/ajh.26203 33891719 PMC8273150

[B74] Wang Y. Fellah S. Fields M. E. Guilliams K. P. Binkley M. M. Eldeniz C. (2021). Cerebral Oxygen Metabolic Stress, Microstructural Injury, and Infarction in Adults with Sickle Cell Disease. Neurology 97, e902–e912. 10.1212/WNL.0000000000012404 34172536 PMC8408504

[B75] Watchmaker J. M. Juttukonda M. R. Davis L. T. Scott A. O. Faraco C. C. Gindville M. C. (2018). Hemodynamic Mechanisms Underlying Elevated Oxygen Extraction Fraction (OEF) in Moyamoya and Sickle Cell Anemia Patients. J. Cereb. Blood Flow Metab. 38, 1618–1630. 10.1177/0271678X16682509 28029271 PMC6125968

[B76] Wechsler D. (2008). Wechsler Adult Intelligence Scale - Fourth Edition (WAIS-IV). San Antonio, 1–3.

[B77] Wu W.-C. Lin S.-C. Wang D. J. Chen K.-L. Li Y.-D. (2013). Measurement of Cerebral White Matter Perfusion Using Pseudocontinuous Arterial Spin Labeling 3T Magnetic Resonance Imaging - an Experimental and Theoretical Investigation of Feasibility. PLoS One 8, e82679. 10.1371/journal.pone.0082679 24324822 PMC3855805

